# Up-regulation of Tissue Factor in Human Pulmonary Artery Endothelial Cells after Ultrafine Particle Exposure

**DOI:** 10.1289/ehp.9556

**Published:** 2007-01-08

**Authors:** Edward D. Karoly, Zhuowei Li, Lisa A. Dailey, Xhevahire Hyseni, Yuh-Chin T. Huang

**Affiliations:** 1 National Health and Environmental Effects Research Laboratory, Office of Research and Development, U.S. Environmental Protection Agency, Research Triangle Park, North Carolina, USA; 2 Center for Environmental Medicine, Asthma and Lung Biology, University of North Carolina, Chapel Hill, North Carolina, USA; 3 Department of Medicine, Duke University Medical Center, Durham, North Carolina, USA

**Keywords:** coagulation cascade, human endothelial cells, microarray, particulate matter, tissue factor

## Abstract

**Background:**

Epidemiology studies have linked exposure to pollutant particles to increased cardiovascular mortality and morbidity, but the mechanisms remain unknown.

**Objectives:**

We tested the hypothesis that the ultrafine fraction of ambient pollutant particles would cause endothelial cell dysfunction.

**Methods:**

We profiled gene expression of human pulmonary artery endothelial cells (HPAEC) exposed to ultrafine particles (UFPs; 100 μg/mL) from Chapel Hill, North Carolina, or vehicle for 4 hr with Affymetrix HG U133 Plus 2.0 chips (*n* = 4 each).

**Results:**

We found 320 up-regulated genes and 106 down-regulated genes (*p* < 0.01, 5% false discovery rate). We noted up-regulation of genes related to coagulation [tissue factor (*F3*) and coagulation factor II receptor-like 2 (*F2RL2*)] and differential regulation of genes related to F3 signaling (*FOS, JUN,* and *NFKBIA*). Results of quantitative polymerase chain reaction show a significant up-regulation of *F3* after 10 and 100 μg/mL UFP exposures. Additionally, the water-soluble fractions of UFPs were sufficient to induce the expression of *F3, F2RL2,* and heme oxygenase 1 (*HMOX1*). Treatment of HPAEC with UFPs for 16 hr increased the release of interleukin (IL)-6 and IL-8. Pretreatment of HPAEC with a blocking antibody against F3 attenuated IL-6 and IL-8 release by 30 and 70%, respectively.

**Conclusions:**

Using gene profiling, we discovered that UFPs may induce vascular endothelial cells to express genes related to clotting. These results indicate that PM may cause adverse cardiovascular health effects by activating coagulation-inflammation circuitry.

Particulate air pollution remains a threat to public health despite the decline in the ambient concentration in the past decades. It has been estimated by the World Health Organization that particulate matter (PM) exposure accounts for 800,000 deaths per year worldwide (Brook et al. 2004). The increased mortality has been attributed to respiratory and more recently to cardiovascular compromise (Borja-Aburto et al. 1998; Brook et al. 2004; Dockery 2001). Short-term exposure to polluted air for as little as 2 hr increases the risk of myocardial infarction in people at risk of developing cardiovascular disease (Peters et al. 2001).

Various mechanisms have been proposed to explain the cardiovascular health effects of PM. These hypotheses include pulmonary and systemic oxidative stress and inflammation (Gurgueira et al. 2002), direct vasoconstriction (Huang et al. 2002; Li et al. 2005a), enhanced coagulation pathways (Nemmar et al. 2002b), and altered cardiac autonomic function (Gold et al. 2000). The ultrafine fraction of ambient pollutant particles [diameter < 100 nm, ultrafine particles (UFPs)] may represent a particular concern because these small particles can remain airborne for an extended period of time. Although they represent only a relatively small fraction of the total mass (Peters et al. 1997), UFPs are a substantial component of PM in the number of particles. Once inhaled, UFPs can be deposited in greater numbers in deeper lung zones than larger particles (Nemmar et al. 2002b) and have a greater potential to permeate the alveolar capillary barrier to be in contact with vascular endothelial cells.

To better understand how UFPs may affect vascular endothelial cells, we profiled gene expression in human pulmonary arterial endothelial cells (HPAEC) exposed to UFPs in Chapel Hill, North Carolina. We hypothesize that UFPs would induce transcriptional activation of genes related to the coagulation and inflammatory response which are signs of endothelial cell dysfunction. Cultured HPAEC were treated with Chapel Hill UFPs (100 μg/mL) or control for 4 hr. RNA from these samples was hybridized to Affymetrix HG U133 Plus 2.0 chips and analyzed for differentially expressed genes. We found that tissue factor (*F3,* Unigene accession no. 62192; http://www.ncbi.nlm.nih.gov/entrez/query.fcgi?db=unigene), coagulation factor II receptor-like 2 (*F2RL2;* Unigene accession no. 42502), interleukin 6 (*IL-6*, Unigene accession no. 512234) and interleukin 8 (*IL-8*; Unigene accession no. 624) were up-regulated. We further showed that the release of IL-6 and IL-8 induced by UFP was partially dependent on the activation of the tissue factor pathway.

## Materials and Methods

### Reagents and chemicals

Molecular mass standards, polyacrylamide, buffers, and protein assay reagents were purchased from Bio-Rad (Richmond, CA). The enhanced chemiluminescent (ECL) blotting detection reagents were purchased from Amersham Biosciences Corp. (Piscataway, NJ). TaqMan Universal PCR master mix and Taqman Gene Expression predeveloped assays (reagents concentrated and preoptimized mixes of primers and FAM-labeled TaqMan probes) for tissue factor (*F3*), coagulation factor II receptor-like 2 (*F2RL2*), coagulation factor VIII associated (*F8A1,* Unigene accession no. 533543; http://www.ncbi.nlm.nih.gov/entrez/query.fcgi?db=unigene), and heme oxygenase 1 (*HMOX1;* Unigene accession no. 517581) were obtained from Applied Biosystems (Foster City, CA). All other chemicals and reagents were from Sigma-Aldrich Co. (St. Louis, MO) unless stated otherwise.

### Collection and extraction of Chapel Hill UFPs

UFPs were collected in July of 2002 in Chapel Hill, North Carolina, outside the U.S. Environmental Protection Agency Human Studies Facility as described previously (Becker et al. 2005). The elemental composition of the UFPs (nanograms per milligram) was described previously with aluminum (948), copper (150), iron (879), lead (181), silicon (1098), and zinc (669) representing the most abundant constituents (Becker et al. 2005). UFP were resuspended in sterile H_2_O (5.0 mg/mL) and stored at −80°C.

### Preparation of the water-soluble and-insoluble fractions of UFP

Water-suspended UFPs were diluted to 25 and 100 μg/mL, centrifuged for 30 min at 20,000 × *g*, and the supernatants were collected as the water-soluble fractions. Pellets from the centrifugation were washed with deionized distilled water 3 times and resuspended in the appropriate volume of water. We designated this as the water-insoluble fraction. Both fractions were stored at −80°C.

### Human pulmonary artery endothelial cells

For microarrays, HPAEC (Cell Applications, Inc., San Diego, CA) were cultured in endothelial growth medium (EGM-2, Clonetics Bio Whittaker Inc., Walkersville, MD), grown to 80% confluency in 100-mm petri dishes, and used during passages 3–5. UFPs were diluted with EGM-2 before experiments. Cells were treated with UFPs (100 μg/mL) or vehicle control for 4 hr at 37°C. For all other experiments, HPAEC were grown to 80% confluency in 6-well plates.

Two different quantitative polymerase chain reaction (Q-PCR) experiments were conducted with UFPs and water-soluble and-insoluble fractions. HPAEC were treated for 4 hr (0, 1, 10, and 100 μg/mL UFP) for *F3* Q-PCR analysis or 2 and 24 hr (0, 25, and 100 μg/mL water-soluble and-insoluble UFP fractions) for all *F3, F2RL2, F8A1,* and *HMOX1*.

### RNA isolation

RNA was extracted with Trizol reagent (GIBCO BRL Life Technologies, Gaithersburg, MD) and purified with Qiagen RNeasy mini columns (Qiagen, Valencia, CA) according to manufacturers’ protocols. Total RNA was resuspended in RNase-free water and assessed with the Agilent 2100 Bioanalyzer and RNA 6000 Nano assay (Agilent Technologies, Palo Alto, CA). All RNA samples had a 28S/18S ratio ≥1.8 and were stored at −80°C before shipment on dry ice to Expression Analysis Inc. (Durham, NC).

### Microarray target preparation and hybridization

RNA target preparation and hybridization to the Affymetrix HG U133 plus 2.0 GeneChip oligonucleotide microarray (Affymetrix, Inc., Santa Clara, CA) was performed by Expression Analysis, Inc. as described previously (Li et al. 2005b). Fluorescent images were detected in a GeneChip Scanner 3000 and expression data was extracted using the default settings in the Microarray Suite 5.0 software (Affymetrix). All GeneChips were scaled to a median intensity setting of 500. For microarray purposes, four biological replicates were collected each for the treated and control samples.

### Q-PCR

Q-PCR was performed for selected genes. cDNAs were synthesized from 0.4 μg of total RNA in 100 μL of buffer containing 5 μM random hexaoligonucleotide primers (Pharmacia, Piscataway, NJ), 10 U/μL Moloney murine leukemia virus reverse transcriptase (GIBCO BRL Life Technologies), 1 U/μL RNase inhibitor (RNasin, Promega, Madison, WI), 0.5 mM dNTP (Pharmacia), 50 mM KCl, 3 mM MgCl_2_, and 10 mM Tris–HCl (pH 9.3) for 1 hr at 39°C. Reverse transcriptase was heat inactivated at 94°C for 4 min.

Q-PCR of specimen and standard cDNA was completed using TaqMan predeveloped assay reagents. Quantitative fluorogenic amplification of cDNA was performed using the ABI Prism 7500 Sequence Detection System, primers and probes of interest and TaqMan Universal PCR Master Mix (Applied Biosystems). The relative abundance of mRNA levels was determined from standard curves generated from a serially diluted standard pool of cDNA prepared from control HPAEC cultures. The relative abundance of glyceraldehyde-3-phosphate dehydrogenase (*GAPDH*, Unigene accession no. 544577; http://www.ncbi.nlm.nih.gov/entrez/query.fcgi?db=unigene) mRNA was used to normalize levels of the mRNAs of interest. For Q-PCR verification experiments, RNA from at least three additional experiments was collected for each timepoint and concentration. RNA samples for microarray and Q-PCR were collected from different experiments and do not represent the same sample.

### Experiments with anti-tissue factor antibody

HPAEC were pretreated with a monoclonal blocking antibody against human F3 (product number 4509; American Diagnostica, Inc., Stamford, CT) (0, 50, and 100 ng/mL) for 15 min with gentle rocking before addition of UFPs (100 μg/mL) or control. UFPs were sonicated for 10 min before addition to cultures. Cell cultures were then incubated for 16 hr at 37°C. Supernatants were removed, centrifuged for 10 min at 150 ×*g* at 4°C, and stored at −80°C. IL-6 and IL-8 protein levels in supernatant samples (R&D Systems, Inc., Minneapolis, MN) were determined by immunoassay according to the manufacturer’s recommendation. Three biological replicates were run in duplicate for each dose and assay.

### Protein isolation and Western blot analysis

After exposure, HPAEC cells were lysed with 500 μL RIPA buffer (1% Nonidet P-40, 0.5% sodium deoxycholate, and 0.1% SDS in phosphate buffered saline (PBS), pH 7.4) containing 1 μL protease inhibitor cocktail (Sigma-Aldrich Co.) per 100 μL RIPA (radioimmunoprecipitation) buffer and sheered with a 22-ga needle. Lysates were centrifuged 10 min at 150 ×*g*, 4°C, and stored at −80°C. Protein concentration of cell lysates was measured with Bio-Rad protein assay reagents according to manufacturer instructions.

For Western blot analysis, cellular proteins were loaded equally, separated by 10% sodium dodecyl sulfate–polyacrylamide gel electrophoresis and transferred to a polyvinylidene difluoride membrane. The blot was then blocked with 5% milk in PBS with 0.05% Tween-20 for 1 hr at room temperature, washed briefly, then probed with a monoclonal antibody against tissue factor (product number 4509; American Diagnostica, Inc.) overnight at 4°C followed by incubation with a horseradish peroxidase–conjugated goat antimouse antibody (Santa Cruz Biotechnology, Santa Cruz, CA). Bands were detected with ECL and films according to the manufacturer instructions (Du Pont-New England Nuclear, Boston, MA). Films were scanned on a ScanJet 6200C with Precision Scan Pro 1.02 (Hewlett Packard, Palo Alto, CA).

### Microarray data analysis

Affymetrix CEL data files were imported into R, an open-source statistical scripting language (http://www.R-project.org; Ihaka and Gentleman 1996) used in conjunction with the Bioconductor project (http://www.bioconductor.org; Gentleman et al. 2004). Normalized values with robust multichip analysis background correction, quantile normalization and median polish were calculated with AffylmGUI (Wettenhall et al. 2006). AffylmGUI allows a graphical user interface for the analysis of Affymetrix microarray GeneChips using the limma package (Smyth 2005) in R. A contrast of treated–control samples was used to generate *p*-values and M-value [log_2_ (treated/control)]. Probe sets with a *p*-value (*p* < 0.01) after an adjustment with a false discovery rate (FDR) 5% were judged by the limma package to be differentially expressed between the treated and control samples. If more than one probe set for the same gene was identified, their M-values (treatment/control ratios) were averaged. The data discussed in this publication have been deposited in the National Center for Biotechnology Information’s Gene Expression Omnibus (GEO; http://www.ncbi.nlm.nih.gov/geo/; Edgar et al. 2002) and are accessible through GEO series accession number GSE4567.

### Biological pathway and network identification

Biological processes represented by the differentially expressed genes were compiled using the Database for Annotation, Visualization and Integrated Discovery [DAVID 2.1 (http://apps1.niaid.nih.gov/david/); Dennis et al. 2003]. Biological pathways were compared to all available *Homo sapiens* pathways specific to the Kyoto Encyclopedia of Genes and Genomes (KEGG 2007). Only pathways with a *p* ≤0.05 calculated by DAVID were retained.

### Statistical analysis for nonmicroarray data

All nonmicroarray data were presented as mean ± SE. A paired *t*-test or a repeated measures analysis of variance (ANOVA) was used to compare differences between treatment and control where appropriate. A *p*-value of *p* < 0.05 was considered to be statistically significant. Statistical analysis was performed with Statview 4.0 (SAS Institute Inc., Cary, NC).

## Results

### Identification of differentially expressed genes associated with UFP exposure

Using the statistical and filtering algorithms described earlier, we identified 664 differentially expressed Affymetrix probe sets. We removed probe sets that lacked an ENTREZ number or were classified as undefined or hypothetical proteins that reduced our list to 426 unique genes, of which 320 were up-regulated and 106 were down-regulated. Using the pathway analysis software, we found three KEGG biological pathways (*p* ≤0.05) overrepresented from the differentially expressed gene list (Tables 1–3). These pathways were the cytokine–cytokine receptor interaction, Wnt signaling, and MAPK signaling. Of note, the cytokine–cytokine interaction pathway contained the most number of genes, many of which have been implicated in the pathogenesis of vascular disease for example, monocyte chemotactit protein 1 (*MCP-1*, Unigene accession no. 303649; http://www.ncbi.nlm.nih.gov/entrez/query.fcgi?db=unigene), *IL-8,* chemokine ligand 1 (*CXCL1,* Unigene accession no. 789), chemokine ligand 2 (*CXCL2,* Unigene accession no. 590921), chemokine ligand 3 (*CXCL3,* Unigene accession no. 89690), and chemokine receptor 4 (*CXCR4,* Unigene accession no. 421986).

We also noted altered expression of several genes related to coagulation. These alterations included up-regulation of tissue factor (*F3*) (2.7-fold) and coagulation factor II receptor-like 2 (*F2RL2*) (1.5-fold) and down-regulation of coagulation factor VIII-associated (intronic transcript) 1 (*F8A1*) (0.8-fold). There were also additional genes associated with the tissue factor (TF)–clotting independent signaling pathway (Table 4).

### Q-PCR and verification of microarray results

**Figure 1:**
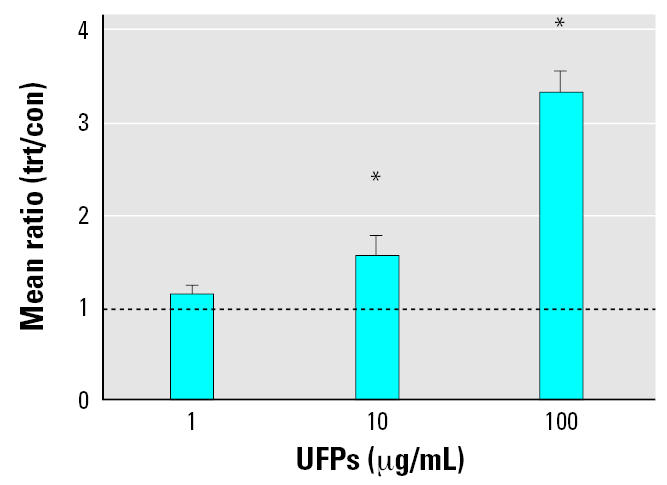
Q-PCR gene expression of tissue factor (*F3*) in cell cultures exposed to UFPs (1, 10, and 100 μg/mL) for 4 hr. *n* = 4 additional experiments for each timepoint and particle exposure. The line indicates a mean ratio of 1.0 or no fold change between treated (trt) and control (con) samples. Error bars indicate mean ± SE. Statistical significance was determined with the paired *t*-test. *Significantly different from control samples, at *p* < 0.05.

The expression of *F3* was verified by Q-PCR. Microarray results showed a 2.7-fold up-regulation in *F3*, whereas Q-PCR showed a 3.3-fold up-regulation. HPAEC were exposed to 0, 1, 10, and 100 μg/mL UFPs *in vitro* for 4 hr and showed significant upregulation in a dose-dependent manner (Figure 1).

**Figure 2:**
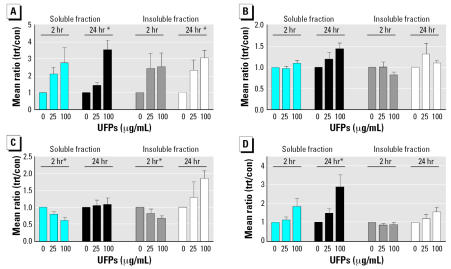
Effect of water-soluble and-insoluble fractions of UFP on gene expression of (*A*) tissue factor (*F3*), (*B*) coagulation factor II receptor-like 2 (*F2RL2*), (*C*) coagulation factor VIII associated 1 (*F8A1*), and (*D*) heme oxygenase (*HMOX1*). Abbreviations: con, control samples; trt, treated samples. Cell cultures were exposed to water-soluble or-insoluble fractions of UFPs (μg/mL) for 2 or 24 hr. mRNA expression was quantified by Q-PCR. *n* = 3 additional experiments for each time point and particle exposure. Error bars indicate mean ± SE. *Significant dose response by repeated measures, ANOVA at *p* < 0.05.

HPAEC were also exposed to water-soluble and-insoluble fractions of UFPs for 2 and 24 hr. Both water-soluble and-insoluble UFP fractions up-regulated the expression of *F3* dose dependently, especially at 24 hr post-exposures (Figure 2A). The water-soluble fraction tended to increase the expression of *F2RL2* (Figure 2B). For heme oxygenase (*HMOX1*), which was upregulated in the microarray (2.5-fold), the water-soluble fraction up-regulated *HMOX1* at 24 hr after UFP exposure (Figure 2D).

*F8A1,* which was down-regulated in the microarray study, showed a significant down-regulation in the Q-PCR results by both soluble and insoluble fractions at 2 hr postexposure (Figure 2C).

### Up-regulation of TF protein expression by UFP

**Figure 3:**
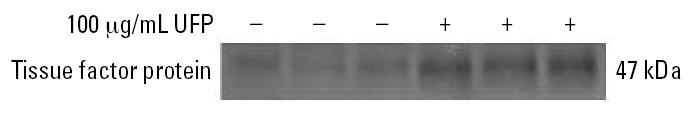
UFP-induced tissue factor protein expression in HPAEC. HPAEC cultures were incubated with 0 or 100 μg/mL UFPs for 16 hr. Fifty micrograms of protein were loaded and measured for tissue factor expression by Western blotting. Results are from three independent experiments.

To determine if the protein expression of TF was up-regulated, HPAEC were incubated with UFP for 18 hr and TF in the cell lysate was measured by Western blot analysis. As shown in Figure 3, UFPs increased TF expression by more than 2-fold (Figure 3).

### Role of TF in UFP-induced cytokine release

**Figure 4:**
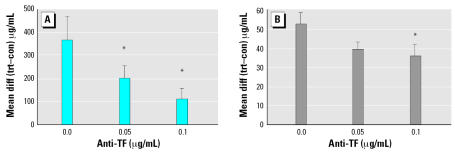
Production of IL-8 (*A*) and IL-6 (*B*) protein expression from HPAEC after exposure to TF-blocking antibody and UFPs. Abbreviations: con, control group; diff, difference; trt, treated group. Cell cultures were preincubated with 0, 0.05, and 0.1 μg/mL TF-blocking antibody followed by 16 hr UFP exposure (100 μg/mL). Values are expressed as the mean difference (diff) between treated (trt) and control (con) samples as measured by ELISA. *n* = 3 independent experiments for each sample. Error bars indicate mean ± SE. *Significant at *p* < 0.05 vs. no antibody by the paired *t*-test.

UFP exposure increased the release of IL-8 by HPAEC by 1.7-fold. Pretreatment with a blocking antibody against TF inhibited the UFP-induced IL-8 release by more than 70% at 0.1 μg/mL of anti-TF (Figure 4A). UFP also increased the release of IL-6 by 1.9-fold. Pretreatment with the blocking antibody against TF attenuated the IL-6 release by approximately 30% at 0.1 μg/mL of anti-TF (*p* = 0.01) (Figure 4B).

## Discussion

Numerous epidemiologic studies have identified a link between PM exposure with adverse cardiovascular outcomes and increased mortality (Borja-Aburto et al. 1998; Dockery et al. 1993). Recent studies have shown a link between PM exposure and oxidative stress, which may impair endothelial-dependent vasodilation as well as increased levels of fibrinogen (an established risk factor for myocardial infarction and stroke), C reactive protein, IL-6, and IL-8 (Brook et al. 2003, 2004). Despite ample evidence of a link between PM exposure and cardiovascular morbidity and mortality, our understanding of the biological mechanisms for these adverse outcomes remains incomplete. Our hypothesis in this study was that the ultrafine fraction of PM, which more likely would have direct contact with pulmonary vasculature when inhaled than fine and course PM, is capable of inducing transcriptional changes indicative of endothelial cell dysfunction. Using microarray analysis, we found 426 unique genes differentially expressed after UFP exposure for 4 hr in HPAEC. Among these were genes related to the coagulation–inflammation circuitry, including up-regulation of *F3*, *F2RL2*, *IL-6,* and *IL-8* and downregulation of *F8A1*. There was also a group of genes that were involved in the pathogenesis of vascular disease, including those associated with the clotting independent signaling of F3, which included v-fos FBJ murine osteosarcoma viral oncogene homolog (*FOS*, Unigene accession no. 25647; http://www.ncbi.nlm.nih.gov/entrez/query.fcgi?db=unigene), v-jun sarcoma virus 17 oncogene homolog (*JUN*, Unigene accession # 525704)*,* nuclear factor kappa-B-cell, subunit 1 (*NFKBIA*, Unigene accession no. 81328), and *HMOX1*. Additionally there also were genes related to the CXC family of chemokines (*MCP-1, IL-8, CXCL1, CXCL2*, *CXCL3,* and *CXCR4*).

In higher organisms, the coagulation cascade is activated to stop the loss of blood after vascular injury by forming a fibrin clot. Inappropriate activation of the coagulation pathways, however, can promote intravascular thrombosis (Chu 2006; Mackman 2005). The blood coagulation cascade is composed of intrinsic and extrinsic pathways. The extrinsic pathway is an inducible signaling cascade that is triggered by up-regulation of TF upon inflammation or endothelial injury (Chu 2006). This initiation process exposes TF to factor VII and forms the tightly bound TF-factor VII–activated complex, which then activates factor X and generates small amounts of thrombin (Busch et al. 2005). The production of thrombin by the extrinsic pathway is critical because it not only generates fibrin from fibrinogen (Davie et al. 1991; Gailani and Broze 1991) but also amplifies the intrinsic pathway. Our study showed that UFPs or their water-soluble fractions induced the gene and protein expression of TF. Together with differential expression of other coagulation-related genes, our *in vitro* study raised the possibility that ambient UFP exposure may activate the coagulation cascade. Preliminary results from our UFP-controlled human exposure study showed a 10–25% increase in indicators of coagulation: prothrombin fragment F1+2 and D-dimers (unpublished observation).

F3 expression is markedly increased within arterial atherosclerotic plaques. Spontaneous plaque rupture may trigger intravascular clotting (Day et al. 2005) and may be a primary cause of thrombus formation in the onset of acute coronary syndromes (Ishibashi et al. 2003). Studies with an *F3*-deficient mouse model suggest that thrombosis after arterial injury is driven primarily by vascular wall F3 expression (Day et al. 2005; Ishibashi et al. 2003). Inactivation of the F3 factor VIIa complex by F3 pathway–inhibitor or active site–blocked factor VIIa has been shown to reduce thrombus weight and increase patency in a rabbit vein injury model (Himber et al. 2002). In our study, UFPs altered the expression of several coagulation-related genes in favor of thrombus formation, that is, up-regulation of *F3*, *F2RL2,* and down-regulation of thrombomodulin (*THBD*, Unigene accession no. 2030; http://www.ncbi.nlm.nih.gov/entrez/query.fcgi?db=unigene). These changes provide biologically plausible mechanisms by which UFPs may promote thrombus formation, namely, activating the TF-mediated extrinsic coagulation pathway and inhibiting the anticoagulant effects of protein C through suppression of thrombomodulin (Nemerson 1988; Van der Meeren et al. 2003).

TF may also produce biological effects via clotting-independent mechanisms (Rickles et al. 2003; Versteeg et al. 2003). After cell activation, the *F3* gene is induced early as a result of the binding of transcription factors, for example, specificity protein-1 (SP-1), activator protein-1 (AP-1), and nuclear factor-kB, to the promoter region of the *F3* gene. The functions of clotting-independent TF pathways are not entirely clear, but regulation of angiogenesis appears to be a major one. TF is known to induce vascular endothelial growth factor (VEGF), one of the most potent regulators of angiogenesis, which in turn up-regulates the expression of TF by activating the early growth response-1 gene (Mechtcheriakova et al. 1999). In tumor-related endothelial cells, decreased PI3-K activity concurrent with increased p38 and ERK1/2 MAPK activity enhances F3 expression by VEGF (Blum et al. 2001). In our study, UFPs altered the expression of many of the genes in these processes, including *FOS* (1.6-fold), *JUN* (1.3-fold), *NFKBIA* (1.3-fold), and early growth response-1 (*EGR-1*, Unigene accession no. 326035; http://www.ncbi.nlm.nih.gov/entrez/query.fcgi?db=unigene) (1.2-fold), indicating potential involvement in activation of clotting-independent mechanisms of TF by UFPs.

Other findings in our study further support the role of PM in the pathogenesis of vascular diseases. For example, we found that UFPs up-regulated genes in the CXC family of chemokines that have been implicated in the pathogenesis of vascular disease. These genes include *MCP-1, IL-8, CXCL1, CXCL2, CXCL3,* and *CXCR4*. MCP-1, which was up-regulated 2.6-fold, has been implicated in the development of vascular disease ranging from arterial injury to the formation of atherosclerosis (Charo and Taubman 2004). Overexpression of MCP-1 in vessel-wall macrophages led to increased foam cells formation and increased atherosclerosis (Aiello et al. 1999). Deletion of MCP-1 in low-density lipoprotein receptor-null mice and mice expressing human apolipoprotein B attenuated the progression of dietary-induced atherosclerosis (Gosling et al. 1999; Gu et al. 1998). Acute coronary syndrome patients with the highest levels of MCP-1 had a significantly increased risk of death or myocardial infarction over 2 months of follow-up (de Lemos et al. 2003). IL-8 also has been linked to the development of early atherosclerotic lesions in the vessels. IL-8, a monocyte agonist, is present in macrophage-rich atherosclerotic plaques (Gerszten et al. 1998, 1999). The expression of these chemokines may enhance the adhesion of circulating monocytes on vascular endothelial cells and participate in atherogenesis (Hagiwara et al. 1998).

It is also well established that there is cross-talk between the coagulation pathways and inflammation in sepsis (Hack 2000; Levi and van der Poll 2005). Infusion of site-inactivated factor VIIa reduced production of cytokines, including IL-6, IL-8 and soluble TNF (tumor necrosis factor) receptor 1 in a baboon model of sepsis (Miller et al. 2002; Welty-Wolf et al. 2001). Genetically modified mice expressing low levels of F3 exhibited reduced IL-6 expression and increased survival during endotoxemia (Pawlinski and Mackman 2004). We now show that there may also be a link between the F3 pathways and inflammation after UFP exposure. This was supported by the findings that the UFP-induced production of IL-6 and IL-8 was attenuated by a blocking antibody against TF. Conversely, TF expression can be induced by inflammatory mediators such as IL-6, IL-8, and MCP-1 (Busch et al. 2005), all of which were up-regulated in our microarray results by 2.30, 2.50, and 2.6-fold, respectively.

It remains controversial whether PM can have direct contact with pulmonary vascular endothelial cells when inhaled. Recent studies have shown that ultrafine carbon black particles may be able to pass from the lung into the systemic circulation (Nemmar et al. 2001, 2002a; Oberdörster et al. 2002), although the mechanisms for the transport of these insoluble particles are unclear. For the UFPs derived from the natural environment, such as those used in our study, the components in the water-soluble fraction may more easily permeate the alveolar capillary barrier via paracellular pathways or metal transporters. Indeed, the water-soluble fractions of UFPs were sufficient to induce the expression of *F3.* The active components in the water-soluble fractions of UFPs, however, are unknown and would require future studies.

In this study, we showed that UFPs at concentrations of 10 μg/mL were sufficient to induce the gene expression of *F3*. These concentrations are higher than the usual ambient level. In Chapel Hill, the ambient UFP concentration is approximately 5,000 particles/cm^3^ (1.6 μg/m^3^) (unpublished observations). The concentration of UFPs, however, may increase 50- to 100-fold during indoor smoking and cooking (Afshari et al. 2005; Dennekamp et al. 2001). If a person works in the latter environment for 4 hr with an average ventilation of 10 L/min, the concentration of UFPs to which alveolar epithelial cells may be exposed *in vivo* may be as high as 10–20 μg/mL, assuming the volume of epithelial lining fluid is 20–40 mL (Rennard et al. 1986). Once UFPs deposit on the lung epithelial cells, the water-soluble fractions of UFPs may reach the endothelial cells and stimulate them to express F3.

In summary, using gene profiling, we found that UFPs induced genes that have been implicated in the pathogenesis of vascular disease, including those linked to clotting-dependent and -independent signaling pathways mediated by TF as well as by the CXC family of chemokines. These results support our hypotheses that UFP exposure may induce endothelial cell dysfunction, leading to vascular changes, and suggest that exposure to high concentrations of UFP may be considered a risk factor for the development of cardiovascular disease.

## Figures and Tables

**Table 1:** Cytokine–cytokine receptor interaction KEGG pathway (*p* = 0.001) identified with DAVID 2.1 from the list of differentially expressed unique genes (*p* < 0.01) (FDR = 0.05).

Accession no.	Gene symbol	Gene name	Fold change
303649	*MCP1*	chemokine (C-C motif) ligand 2	2.6
624	*IL8*	interleukin 8	2.5
89690	*CXCL3*	chemokine (C-X-C motif) ligand 3	2.3
512234	*IL6*	interleukin 6	2.3
396530	*HGF*	hepatocyte growth factor	2.2
590921	*CXCL2*	chemokine (C-X-C motif) ligand 2	2.0
204044	*TNFRSF11A*	tumor necrosis factor receptor, superfamily member 11A	1.8
389874	*TSLP*	thymic stromal lymphopoietin	1.6
557403	*IL1R1*	interleukin-1 receptor, type I	1.6
513457	*IL4R*	interleukin 4 receptor	1.5
2250	*LIF*	leukemia inhibitory factor	1.5
789	*CXCL1*	chemokine (C-X-C motif) ligand 1	1.4
28792	*INHBA*	inhibin, beta a	1.4
479756	*KDR*	kinase insert domain receptor	1.3
438918	*ACVR1B*	activin A receptor, type IB	1.3
421986	*CXCR4*	chemokine (C-X-C motif) receptor 4	1.3
1048	*KITLG*	kit ligand	1.3
1976	*PDGFB*	platelet-derived growth factor beta polypeptide	0.8
256278	*TNFRSF1B*	tumor necrosis factor receptor, superfamily member 1B	0.8

Gene annotations are from Unigene (http://www.ncbi.nlm.nih.gov/entrez/query.fcgi?db=unigene).

**Table 2:** Wnt signaling KEGG pathway (*p* = 0.02) identified with DAVID 2.1 from the list of differentially expressed unique genes (*p* < 0.01) (FDR = 0.05).

Accession no.	Gene symbol	Gene name	Fold change
211869	*DKK2*	dickkopf homolog 2	1.9
592141	*NFATC2*	nuclear factor of activated T-cells, cytoplasmic, calcineurin-dependent 2	1.8
599590	*SOX17*	SRY (sex determining region Y)-box 17	1.4
6347	*LRP5*	low density lipoprotein receptor-related protein 5	1.3
525704	*JUN*	v-jun sarcoma virus 17 oncogene homolog	1.3
283565	*FOSL1*	fos-like antigen 1	1.2
351887	*CAMK2B*	calcium/calmodulin-dependent protein kinase II beta	1.2
149413	*PPP3CC*	protein phosphatase 3, catalytic subunit, gamma	1.2
		isoforms (calcineurin A gamma)	
94234	*FZD1*	frizzled homolog 1	0.8
591968	*FZD4*	frizzled homolog 4	0.8
524348	*PRICKLE1*	prickle-like 1	0.8

Gene annotations are from Unigene (http://www.ncbi.nlm.nih.gov/entrez/query.fcgi?db=unigene).

**Table 3:** MAPK signaling KEGG pathway (*p* = 0.05) identified with DAVID 2.1 from the list of differentially expressed unique genes (*p* < 0.01) (FDR = 0.05).

Accession no.	Gene symbol	Gene name	Fold change
417962	*DUSP4*	dual specificity phosphatase 4	1.7
510225	*RPS6KA5*	ribosomal protein S6 kinase, polypeptide 5	1.6
25647	*FOS*	v-fos FBJ murine osteosarcoma viral oncogene homolog	1.6
557403	*IL1R1*	interleukin-1 receptor, type I	1.6
432453	*MAP3K8*	mitogen-activated protein kinase kinase kinase 8	1.5
497200	*PLA2G4A*	phospholipase A2, group IVA	1.5
278733	*SOS1*	son of sevenless homolog 1	1.3
438918	*ACVR1B*	activin A receptor, type IB	1.3
525704	*JUN*	v-jun sarcoma virus 17 oncogene homolog	1.3
149413	*PPP3CC*	protein phosphatase 5 catalytic subunit, gama isoform	1.2
390428	*MAP3K4*	mitogen-activated protein kinase kinase kinase 4	0.9
1976	*PDGFB*	viral (v-sis) oncogene homolog	0.8
186486	*MAP3K5*	mitogen-activated protein kinase kinase kinase 5	0.8

Gene annotations are from Unigene (http://www.ncbi.nlm.nih.gov/entrez/query.fcgi?db=unigene).

**Table 4:** Tissue factor clotting–independent mechanism associated genes from the list of differentially expressed unique genes (*p* < 0.01) (FDR = 0.05).

Accession no.	Gene symbol	Gene name	Fold change
517617	*MAFF*	v-maf musculoaponeurotic fibrosarcoma oncogene homolog F	1.7
25292	*JUNB*	jun B proto-oncogene	1.7
590958	*FOSB*	FBJ murine osteosarcoma viral oncogene homolog B	1.5
132225	*PIK3R1*	phosphoinositide-3-kinase, regulatory subunit 1	1.5
155396	*NRF2*	nuclear factor (erythroid-derived 2)-like 2	1.3
534313	*EGR3*	early growth response 3	1.3
81328	*NFKBIA*	nuclear factor of kappa light polypeptide gene enhancer in B-cells	1.3
		inhibitor, alpha	
252229	*MAFG*	v-maf musculoaponeurotic fibrosarcoma oncogene homolog G (avian)	1.2
326035	*EGR1*	early growth response 1	1.2

Gene annotations are from Unigene (http://www.ncbi.nlm.nih.gov/entrez/query.fcgi?db=unigene).
